# Ro-vibrational energies of cesium dimer and lithium dimer with molecular attractive potential

**DOI:** 10.1038/s41598-021-85761-x

**Published:** 2021-03-18

**Authors:** C. A. Onate, T. A. Akanbi, I. B. Okon

**Affiliations:** 1 Department of Physical Sciences, Landmark University, Omu-Aran, Nigeria; 2 Theoretical Physics Group, Department of Physics, University of Uyo, Uyo, Nigeria

**Keywords:** Mathematics and computing, Physics

## Abstract

An approximate solution of the Schrӧdinger equation for a molecular attractive potential was obtained using the parametric Nikiforov–Uvarov method. The energy equation and the corresponding radial wave functions were calculated. The effects of the potential parameters on the energy eigenvalues were examined. The thermal properties under the molecular attractive potential were calculated and the behaviour of the thermal properties with the maximum quantum state (λ) and the temperature parameter (β) respectively, were studied. Using the molecular spectroscopic parameters, the Rydberg–Klein–Rees (RKR) of cesium dimer and lithium dimer were both obtained and compared with the experimental values. The RKR values of both cesium dimer and lithium dimer calculated aligned with the observed values. The deviation and average deviation of the RKR for each molecule were also calculated.

## Introduction

A good understanding of a molecular structure depends on the internuclear and molecular potential models. This creates a larger space of study on the molecular potential functions in the diatomic domain, which geared different authors to carry out a number of researches in this area. The molecular potential functions have spectroscopic parameters such as the dissociation energy $(D_{e} ),$ equilibrium bond separation $(r_{e} )$ and a screening parameter $(\alpha )$ that make them suitable for the description of the diatomic molecules. The molecular screening parameter is calculated using a simple formula^1–3^

1 $$\alpha = \pi c\omega_{e} \sqrt {\frac{2\mu }{{D_{e} }}} + \frac{1}{{r_{e} }}W\left( {\pi c\omega_{e} r_{e} \sqrt {\frac{2\mu }{{D_{e} }}} e^{{ - \pi c\omega_{e} r_{e} \sqrt {\frac{2\mu }{{D_{e} }}} }} } \right),$$

where $\omega_{e}$ is a vibrational frequency, $c$ is speed of light, $W$ is Lambert function, $\mu$ is the reduced mass. With the aid of the spectroscopic parameters, several molecular potential functions were formulated and studied for different applications. For instance, the improved Rosen Morse potential function formulated by Jia et al.^4^, was used to study the RKR of sodium dimer, nitrogen dimer and cesium dimer respectively in Refs.^2,5,6^. In Refs.^7^ and^8^ respectively, the improved Manning-Rosen potential function also formulated by Jia et al.^4^ was used to calculate the rotation vibrational transition frequency of hydrogen fluoride (HF). The Tietz-Wei potential function was used to study eigensolutions and thermodynamic properties in Ref.^9^. In Ref.^3^, an improved deformed four parameter exponential-type potential model was formulated and studied under the RKR of cesium molecule. The same RKR of cesium molecule was studied under Tietz-Hua oscillator in Ref.^10^. Desai et al.^11^, in one of their papers, calculated the RKR of nitrogen molecule and hydrogen molecule under the Morse potential and modified Morse potential. Recently, Horchani et al.^12^, formulated an improved generalized Pӧschl–Teller oscillator and obtained the RKR of potassium molecule. Jia et al.^13^, in one of the papers, calculated the thermodynamic properties of lithium dimer under the improved Manning-Rosen potential. Onate and Idiodi^14^, reported Fisher information and complexity measures of the generalized Morse potential model. The thermodynamic properties of Shifted Deng-Fan potential was also reported by Oyewumi et al.^15^. The scattering state solutions for Manning-Rosen potential was reported by Qiang et al.^16^. In Ref.^17^, Idiodi and Onate obtained Fisher information and variance for Frost Musulin potential. Onate et al.^18^, also reported some theoretic quantities under Tietz-Hua potential. Edet et al.^19^, calculated the thermal properties of the Deng–Fan–Eckart potential. The bound state solutions and energy spectra for some molecules under some molecular potentials were also studied and reported by different authors. Motivated by the usefulness and influence of the molecular potentials, this study aims to transform an attractive potential model to a molecular potential model and study the one-dimensional Schrödinger equation with the transform potential. The study also intends to calculate the RKR values of two molecules (cesium dimer and lithium dimer) and thermodynamic properties of the potential. The attractive potential is a four-parameter exponential-type potential formulated in 1993 by Williams and Pouliss^20^. According to these authors, the four parameters are $A,$ $B,$ $C$ and $\alpha .$ The attractive potential has received acceptable reports under relativistic and non-relativistic regime by few authors^21–23^. The potential cannot be used to describe the detail features of a molecule since there are no spectroscopic parameters. Here, the attractive potential will be modified into a molecular potential function to fit the description of a given molecule. The molecular attractive potential model in this study is given by

2 $$V(r) = D_{e} \left[ {\frac{{Ae^{{2\alpha r_{e} }} + Be^{{\alpha (r + r_{e} )}} + Ce^{2\alpha r} }}{{(1 - e^{ - \alpha r} )^{2} }}} \right]e^{ - 2\alpha r} .$$

The parameters $A,$ $B$ and $C$ are potential parameters. In the previous reports on the attractive potential, the potential parameters were given as $A = \left( {\frac{\alpha }{2}} \right)^{2} ,$ $B = \frac{{\lambda_{0} \alpha^{2} }}{4} - 2\alpha^{2}$ and $C = \alpha^{2} - \frac{{\lambda_{0} \alpha^{2} }}{4},$ where $- \infty < \lambda_{0} < + \infty ,$ however, in our computations, these values may not be used. In the present work, these parameters are given as $A = \frac{{\lambda_{0} }}{2},$ $B = 2(1 - \lambda_{0} )$ and $C = A + B + 2.$ This is to enable us have the desired results. The scheme of our work is as follows: The bound state of the radial Schrödinger equation is given in the next section. The thermal properties of the potential are given in section 3. The discussion of results and conclusion respectively are given in sections 4 and 5.

## Bound state solutions of the Schrödinger equation with molecular attractive potential

From the three-dimensional Schrödinger equation, the radial Schrödinger equation with a centrifugal term is given as

3 $$- \frac{{\hbar^{2} }}{2\mu }\frac{{d^{2} R_{n\ell } (r)}}{{dr^{2} }} + \frac{{\hbar^{2} }}{2\mu }\frac{\ell (\ell + 1)}{{r^{2} }}R_{n\ell } (r) = E_{n\ell } R_{n\ell } (r) - V(r)R_{n\ell } (r),$$

where $V(r)$ is the interacting potential, $E_{n\ell }$ is the non-relativistic energy of the system, $\hbar$ is the reduced Planck’s constant, $n$ is the quantum number,$R_{n\ell } (r)$ is the wave function. The centrifugal term in Eq. (3) can be over-come by employing the following approximation scheme

4 $$\frac{1}{{r^{2} }} \approx \alpha^{2} \frac{{e^{ - \alpha r} }}{{(1 - e^{ - \alpha r} )^{2} }}.$$

In this work, the authors decided to use parametric Nikiforov–Uvarov method for the calculation of the energy equation and the wave functions. The parametric Nikiforov–Uvarov method is a popular and accurate method to obtain energy equation and the wave functions. It was derived from the original Nikiforov–Uvarov method^25^. The method has been widely reported and as such, the detail of the method will not be presented in this work. To use this method, Tezcan and Sever^26^, formulated a general form of a second-order differential equation of the form

5 $$\left( {\frac{{d^{2} }}{{ds^{2} }} + \frac{{\alpha_{1} - \alpha_{2} s}}{{s(1 - \alpha_{3} s)}}\frac{d}{ds} + \frac{{ - \xi_{1} s^{2} + \xi_{2} s - \xi_{3} }}{{s^{2} (1 - \alpha_{3} s)^{2} }}} \right)\psi (s) = 0.$$

Following the work of these authors, the condition for eigenvalues equation and wave functions respectively, are given by^24,26–29^

6 $$n\alpha_{2} - \left( {2n + 1} \right)\alpha_{5} + \alpha_{7} + 2\alpha_{3} \alpha_{8} + n\left( {n - 1} \right)\alpha_{3} + \left( {2n + 1} \right)\sqrt {\alpha_{9} } + \left( {2\sqrt {\alpha_{9} } + \alpha_{3} \left( {2n + 1} \right)} \right)\sqrt {\alpha_{8} } = 0,$$

7 $$\psi (s) = Ns^{{\alpha_{12} }} (1 - \alpha_{3} s)^{{ - \alpha_{12} - \frac{{\alpha_{13} }}{{\alpha_{3} }}}} \times P_{n}^{{\left( {\alpha_{10} - 1,\frac{{\alpha_{11} }}{{\alpha_{3} }} - \alpha_{10} - 1} \right)}} (1 - 2\alpha_{3} s),$$

where $P_{n}^{(\alpha ,\beta )}$ are Jacobi polynomials. The parametric constants in Eqs. (6) and (7) are mathematically deduced as

8 $$\left. \begin{gathered} \alpha_{4} = \frac{{1 - \alpha_{1} }}{2},\alpha_{5} = \frac{{\alpha_{2} - 2\alpha_{3} }}{2},\alpha_{6} = \alpha_{5}^{2} + \xi_{1} ,\alpha_{7} = 2\alpha_{4} \alpha_{5} - \xi_{2} ,\alpha_{8} = \alpha_{4}^{2} + \xi_{3} , \hfill \\ \alpha_{9} = \alpha_{3} \left( {\alpha_{7} + \alpha_{3} \alpha_{8} } \right) + \alpha_{6} ,\alpha_{10} = \alpha_{1} + 2\alpha_{4} + 2\sqrt {\alpha_{8} } ,\alpha_{11} = \alpha_{2} - 2\alpha_{5} + 2\left( {\sqrt {\alpha_{9} } + \alpha_{3} \sqrt {\alpha_{8} } } \right), \hfill \\ \alpha_{12} = \alpha_{4} + \sqrt {\alpha_{8} } ,\alpha_{13} = \alpha_{5} - \left( {\sqrt {\alpha_{9} } + \alpha_{3} \sqrt {\alpha_{8} } } \right) \hfill \\ \end{gathered} \right\}.$$

The detail of the methodology can be found in Ref.^26^ and other literatures. When Eq. (2) and Eq. (4) are substituted into Eq. (3), the radial Schrödinger equation given in Eq. (3) becomes

9 $$\frac{{d^{2} R_{n,\ell } (r)}}{{dr^{2} }} + \left[ {\frac{2\mu }{{\hbar^{2} }}\left( {E_{n,\ell } - D_{e} \left[ {\frac{{Ae^{{2\alpha r_{e} }} + Be^{{\alpha (r + r_{e} )}} + Ce^{2\alpha r} }}{{(1 - e^{ - \alpha r} )^{2} }}} \right]e^{ - 2\alpha r} } \right) - \frac{{\ell (\ell + 1)\alpha^{2} e^{ - \alpha r} }}{{(1 - e^{ - \alpha r} )^{2} }}} \right]R_{n,\ell } (r)  = 0.$$

Defining a variable of the form $y = e^{ - \alpha r} ,$ and substitute it into Eq. (9), then, we have

10 $$\frac{{d^{2} R_{n,\ell } (y)}}{{dy^{2} }} + \frac{1 - y}{{y(1 - y)}}\frac{{dR_{n,\ell } (y)}}{dy} + \left[ {\frac{{ - (\varepsilon Ae^{{2\alpha r_{e} }} - \delta_{0} )y^{2} - (2\delta_{0} + \varepsilon Be^{{\alpha r_{e} }} + \ell (\ell + 1))y - \varepsilon C + \delta_{0} }}{{y^{2} (1 - y)^{2} }}} \right]R_{n,\ell } (y) = 0,$$

where

11 $$\left. {\delta_{0} = \frac{{2\mu E_{n,\ell } }}{{\alpha^{2} \hbar^{2} }},\varepsilon = \frac{{2\mu D_{e} }}{{\alpha^{2} \hbar^{2} }}} \right\}.$$

Comparing Eq. (10) with Eqs. (5), (8) turns out to be

12 $$\left. \begin{gathered} \alpha_{1} = \alpha_{2} = \alpha_{3} = 1,\alpha_{4} = 0,\alpha_{5} = - \frac{1}{2},\alpha_{6} = \frac{1}{4} - \delta_{0} + \varepsilon Ae^{{2\alpha r_{e} }} ,\alpha_{7} = \ell (\ell + 1) + \varepsilon Be^{{\alpha r_{e} }} + 2\delta_{0} , \hfill \\ \alpha_{8} = \varepsilon C - \delta_{0} ,\alpha_{9} = \frac{1}{4} + \varepsilon (Ae^{{2\alpha r_{e} }} + Be^{{\alpha r_{e} }} + C) + \ell (\ell + 1),\alpha_{10} = 1 + 2\sqrt {\varepsilon C - \delta_{0} } , \hfill \\ \alpha_{11} = 2\left( {1 + \sqrt {\varepsilon C - \delta_{0} } } \right) + \sqrt {(1 + 2\ell )^{2} + 4\varepsilon (Ae^{{2\alpha r_{e} }} + Be^{{\alpha r_{e} }} + C)} ,\alpha_{12} = \sqrt {\varepsilon C - \delta_{0} } , \hfill \\ \alpha_{13} = - \frac{1}{2} - \frac{1}{2}\sqrt {(1 + 2\ell )^{2} + 4\varepsilon (Ae^{{2\alpha r_{e} }} + Be^{{\alpha r_{e} }} + C)} - \sqrt {\varepsilon C - \delta_{0} } \hfill \\ \end{gathered} \right\}.$$

Substituting Eq. (12) into Eqs. (6) and (7) respectively, we have the energy equation and its corresponding wave functions for the system as follows

13 $$E_{n,\ell } = CD_{e} - \frac{{\alpha^{2} \hbar^{2} }}{2\mu }\left[ {\frac{{\varepsilon (2C + Be^{{\alpha r_{e} }} ) + \ell (\ell + 1) + n(n + 1) + \frac{1}{2} + \left( {n + \frac{1}{2}} \right)\vartheta }}{1 + 2n + \vartheta }} \right]^{2} ,$$

14 $$R_{n,\ell } (y) = y^{\chi } (1 - y)^{{\frac{1}{2}\left( {1 + \vartheta } \right)}} \times P_{n}^{{\left( {2\chi ,\vartheta } \right)}} (1 - 2y),$$

15 $$\chi = \sqrt {\varepsilon C - \frac{{2\mu E_{n,\ell } }}{{\alpha^{2} \hbar^{2} }}} ,$$

16 $$\vartheta = \sqrt {(1 + 2\ell )^{2} + 4\varepsilon (Ae^{{2\alpha r_{e} }} + Be^{{\alpha r_{e} }} + C)} .$$

## The molecular attractive potential and thermodynamic properties

To calculate the thermodynamic properties for the molecular attractive potential, the energy equation given in Eq. (13) is written in a compact form which is purely vibrational. Thus Eq. (13) is written as

17 $$E_{n} = - \left[ {Q_{2} \rho^{2} + \frac{{Q_{2} Q_{3}^{2} }}{{\rho^{2} }}} \right] + Q_{1} - 2Q_{2} Q_{3} ,$$

18 $$\left. \begin{gathered} Q_{1} = D_{e} ,\delta = \frac{1}{2} + \frac{1}{2}\sqrt {1 + 4\varepsilon \left( {Ae^{{2\alpha r_{e} }} + Be^{{\alpha r_{e} }} + C} \right)} , \hfill \\ Q_{2} = \frac{{\alpha^{2} \hbar^{2} }}{8\mu },Q_{3} = - \varepsilon Ae^{{2\alpha r_{e} }} \hfill \\ \end{gathered} \right\}.$$

Having written the energy equation in a compact form that is suitable for the calculation of the thermodynamic properties, then, the partition function of the system can be define as

19 $$z = \int\limits_{0}^{\lambda } {e^{{ - \beta E_{n} }} dn} .$$

Now, defining $\rho = \left( {n + \delta } \right)$ and substituting Eqs. (17) into (19), the partition function given above can easily be written in the form

20 $$z = e^{{\beta \left( {2Q_{2} Q_{3} - Q_{1} } \right)}} \int\limits_{0}^{\lambda } {e^{{\beta \left( {Q_{2} \rho^{2} + \frac{{Q_{2} Q_{3}^{2} }}{{\rho^{2} }}} \right)}} d\rho } ,$$

where $\lambda_{\max } = - \delta + \sqrt {Q_{3} } .$ Using Mathematica 10.0 version, the partition function of Eq. (20) becomes

21 $${\rm Z}\left( \beta \right) = \frac{{e^{{\chi_{1} }} \left[ {e^{{ - \chi_{2} }} \left( {1 + \chi_{4} } \right) + e^{{\chi_{3} }} \left( { - 1 + \chi_{5} } \right)} \right]}}{{4\sqrt { - \beta Q_{2} } }},$$

where

22 $$\left. \begin{gathered} \chi_{1} = \beta \left( { - Q_{1} + 2Q_{2} Q_{3} } \right),\chi_{2} = 2\sqrt { - \beta Q_{2} } \sqrt { - \beta Q_{2} Q_{3}^{2} } ,\chi_{3} = - 2\sqrt { - \beta Q_{2} } \sqrt { - \beta Q_{2} Q_{3}^{2} } , \hfill \\ \chi_{4} = erf\left[ {\lambda \sqrt { - \beta Q_{2} } - \frac{{\sqrt { - \beta Q_{2} Q_{3}^{2} } }}{\lambda }} \right],\chi_{5} = erf\left[ {\lambda \sqrt { - \beta Q_{2} } + \frac{{\sqrt { - \beta Q_{2} Q_{3}^{2} } }}{\lambda }} \right] \hfill \\ \end{gathered} \right\}.$$

### Vibrational mean energy

23 $$\begin{gathered} U\left( \beta \right) = - \frac{\partial \ln Z\left( \beta \right)}{{\partial \beta }} \Rightarrow \hfill \\ = U\left( \beta \right) = - \left\{ {\frac{{\left[ \begin{gathered} 1 + \chi_{4} + e^{{2\chi_{2} }} \left( { - 1 + \chi_{5} } \right) - 2\beta \left[ {1 + \chi_{4} + e^{{2\chi_{2} }} \left( { - 1 + \chi_{5} } \right)} \right]\Lambda_{0} + \hfill \\ \Lambda_{1} 4\sqrt { - \beta Q_{2} } \left[ {\sqrt \pi \beta \left( { - 1 - e^{{2\chi_{2} }} - \chi_{4} + e^{{2\chi_{2} }} \chi_{5} } \right)Q_{2} Q_{3}^{2} - e^{{\chi_{2} + \Lambda_{2} }} } \right] \hfill \\ \end{gathered} \right]}}{{2\beta \left[ {1 + \chi_{4} + e^{{2\chi_{2} }} \left( { - 1 + \chi_{5} } \right)} \right]}}} \right\}, \hfill \\ \end{gathered}$$

where

24 $$\left[ {\Lambda_{0} = \left( { - Q_{1} + 2Q_{2} Q_{3} } \right)\begin{array}{*{20}c} , & {\Lambda_{1} = \frac{1}{{\sqrt \pi \sqrt { - \beta Q_{2} Q_{3}^{2} } }}\begin{array}{*{20}c} , & {\Lambda_{2} = \frac{{\beta Q_{2} \left( {\lambda^{4} + Q_{3}^{2} } \right)}}{{\lambda^{2} }}\lambda \sqrt { - \beta Q_{2} Q_{3}^{2} } } \\ \end{array} } \\ \end{array} } \right].$$

### Vibrational specific heat capacity

25 $$C\left( \beta \right) = K\beta^{2} \frac{{\partial^{2} \ln Z\left( \beta \right)}}{{\partial \beta^{2} }} = \frac{{\Delta_{0} + \Delta_{1} - 8\Delta_{2} + \Delta_{3} }}{{2\pi \lambda \left( {1 - e^{{2\chi_{2} }} + \chi_{4} + e^{{2\chi_{2} }} \chi_{5} } \right)^{2} \Lambda_{3} }},$$

where

26 $$\left. \begin{gathered} \Delta_{0} = \pi \lambda \left( {1 - e^{{2\chi_{2} }} + \chi_{4} + e^{{2\chi_{2} }} \chi_{5} } \right)^{2} \Lambda_{3} + 2e^{{\chi_{2} + \Lambda_{2} }} \beta \lambda^{2} Q_{2} \left[ \begin{gathered} \sqrt \pi - e^{{2\chi_{2} }} \sqrt \pi + \sqrt \pi \chi_{4} \hfill \\ + e^{{2\chi_{2} }} \sqrt \pi \chi_{5} + 4e^{{\chi_{2} + \Lambda_{2} }} \lambda \sqrt { - \beta Q_{2} } \hfill \\ \end{gathered} \right]\Lambda_{6} , \hfill \\ \Delta_{1} = 4e^{{\chi_{2} }} \sqrt \pi \beta^{2} Q_{{_{2} }}^{2} \left[ { - e^{{\Lambda_{4} }} \lambda^{4} \left( {1 - e^{{2\chi_{2} }} + \chi_{4} + e^{{2\chi_{2} }} \chi_{5} } \right)} \right]\Lambda_{6} + Q_{3}^{2} \left( {4e^{{\Lambda_{2} }} \Lambda_{5} + 4e^{{2\chi_{2} + \Lambda_{2} }} \Lambda_{5} + e^{{\Lambda_{2} }} \Lambda_{6} - e^{{2\chi_{2} + \Lambda_{2} }} \Lambda_{6} } \right), \hfill \\ \Delta_{2} = e^{{\chi_{2} }} \sqrt \pi \lambda \Lambda_{3} + \Delta_{4} e^{{\chi_{2} }} \chi_{5} \left[ {8\sqrt \pi \lambda \Lambda_{3} + e^{{\chi_{2} + \Lambda_{2} }} \left( { - 4\lambda^{2} \sqrt { - \beta Q_{2} } + \sqrt { - \beta Q_{2} Q_{3}^{2} } } \right)} \right],\Lambda_{3} = \sqrt { - \beta Q_{2} } \sqrt { - \beta Q_{2} Q_{3}^{2} } , \hfill \\ \Delta_{3} = \chi_{4} \left[ { - 8e^{{\chi_{2} }} \sqrt \pi \lambda \Lambda_{3} + 8e^{{\chi_{2} }} \sqrt \pi \lambda \Lambda_{3} + e^{{\Lambda_{2} }} \left( {4\lambda^{2} \Lambda_{3} } \right)} \right],\Lambda_{4} = \frac{{\beta Q_{2} \left( {\lambda^{4} + Q_{3}^{2} } \right)}}{{\lambda^{2} }},\Lambda_{5} = \lambda^{2} \sqrt { - \beta Q_{2} } ,\Lambda_{6} = \sqrt { - \beta Q_{2} Q_{3}^{2} } , \hfill \\ \end{gathered} \right\}.$$

### Vibrational entropy

27 $$\begin{gathered} = k\ln Z\left( \beta \right) - k\beta \frac{\ln Z\left( \beta \right)}{{\partial \beta^{2} }} = k\ln \left[ {\frac{{e^{{ - \beta Q_{1} + 2\beta Q_{1} - \chi_{2} }} \sqrt \pi \left[ {1 + \chi_{4} + e^{{2\chi_{2} }} \left( {\chi_{5} + 1} \right)} \right]}}{{4\sqrt { - \beta Q_{2} } }}} \right] \hfill \\ - K\beta \left\{ {\frac{{\left[ \begin{gathered} 1 + \chi_{4} + e^{{2\chi_{2} }} \left( {\chi_{5} - 1} \right) - 2\beta \left( {1 + \chi_{4} + e^{{2\chi_{2} }} \left( {\chi_{5} + 1} \right)} \right)\Lambda_{0} \hfill \\ + \left[ {\frac{{4\sqrt { - \beta Q_{2} } \left( {\sqrt \pi \beta \left( {e^{{2\chi_{2} }} \chi_{5} - e^{{2\chi_{2} }} - \chi_{4} - 1} \right)Q_{2} Q_{3}^{2} - e^{{\chi_{2} + \Lambda_{2} \Lambda_{6} }} } \right)}}{{\sqrt \pi \Lambda_{6} }}} \right] \hfill \\ \end{gathered} \right]}}{{2\beta \left[ {1 + \chi_{4} + e^{{2\chi_{2} }} \left( {\chi_{5} - 1} \right)} \right]}}} \right\}. \hfill \\ \end{gathered}$$

### Vibrational free energy

28 $$F(\beta ) = - \frac{1}{\beta }\ln Z\left( \beta \right) = - \frac{{e^{{\beta \Lambda_{0} }} \ln \sqrt \pi \left[ {e^{{\chi_{3} }} \left( {\chi_{4} + 1} \right) + e^{{\chi_{2} }} \left( {\chi_{5} - 1} \right)} \right]}}{{4\beta \sqrt { - \beta Q_{2} } }}.$$

## Discussion

**Figure 1:**
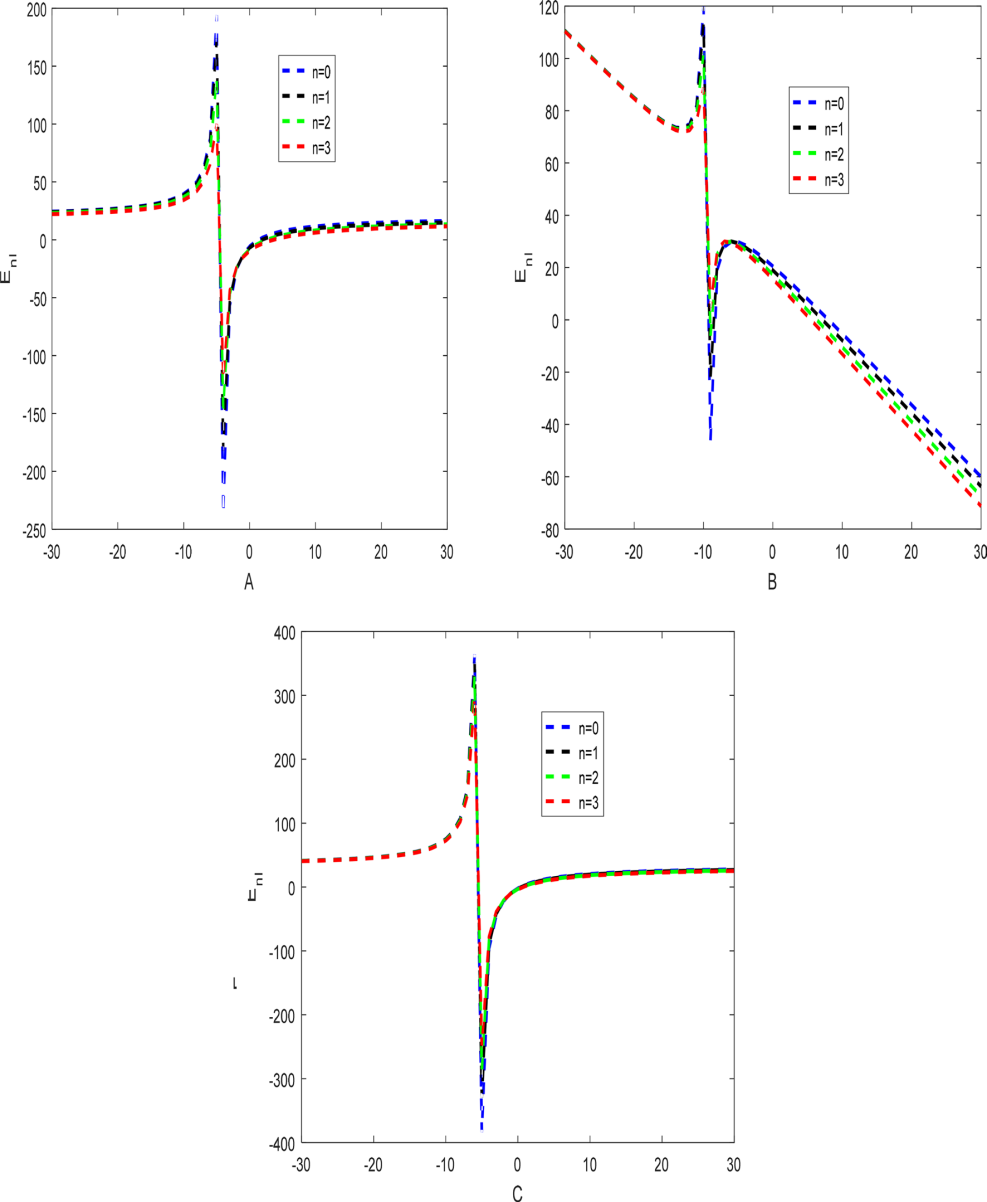
Variation of energy against (**A**,**B**,**C**) respectively.

**Figure 2:**
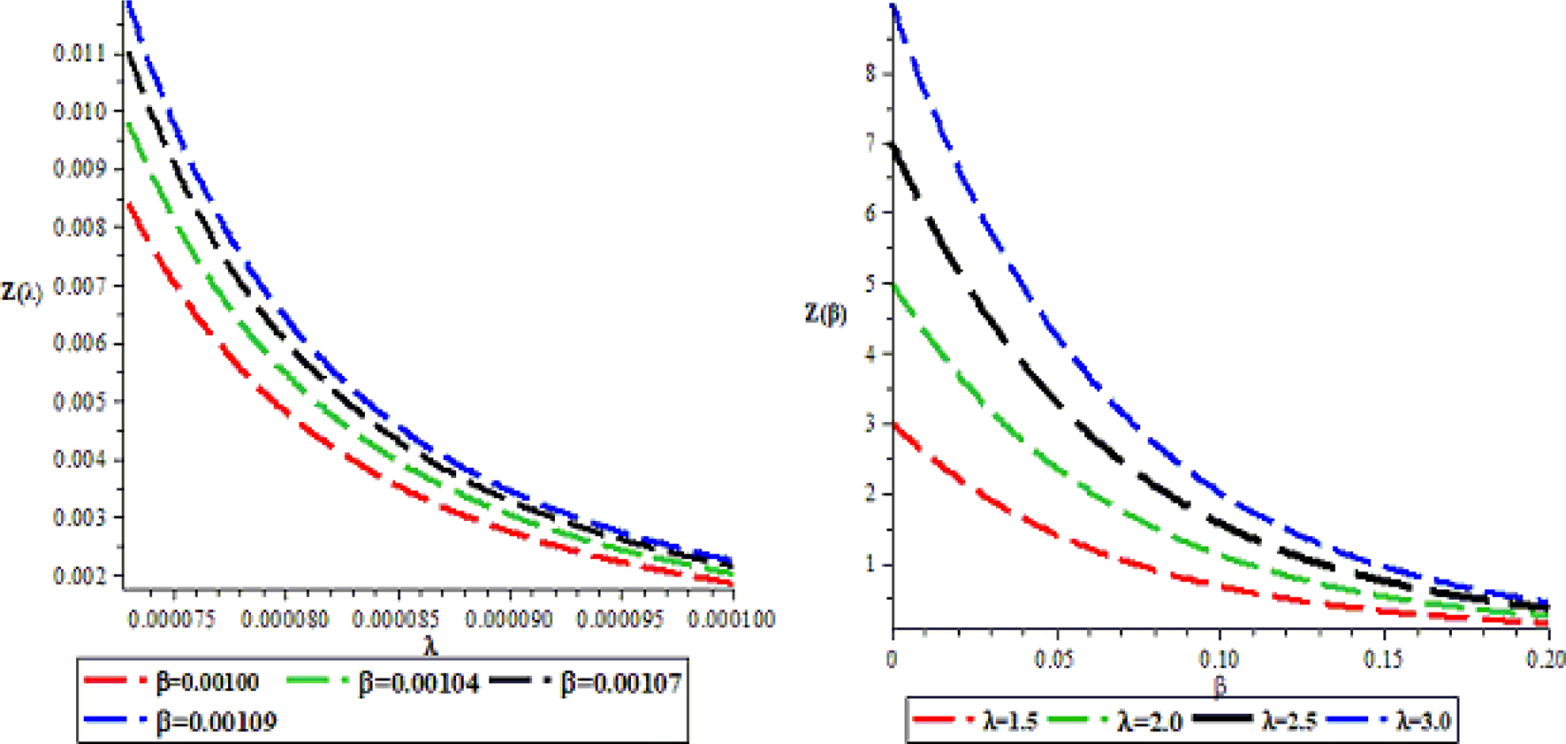
The variation of vibrational partition function against the maximum quantum state (λ) and temperature parameter (β) respectively.

**Figure 3:**
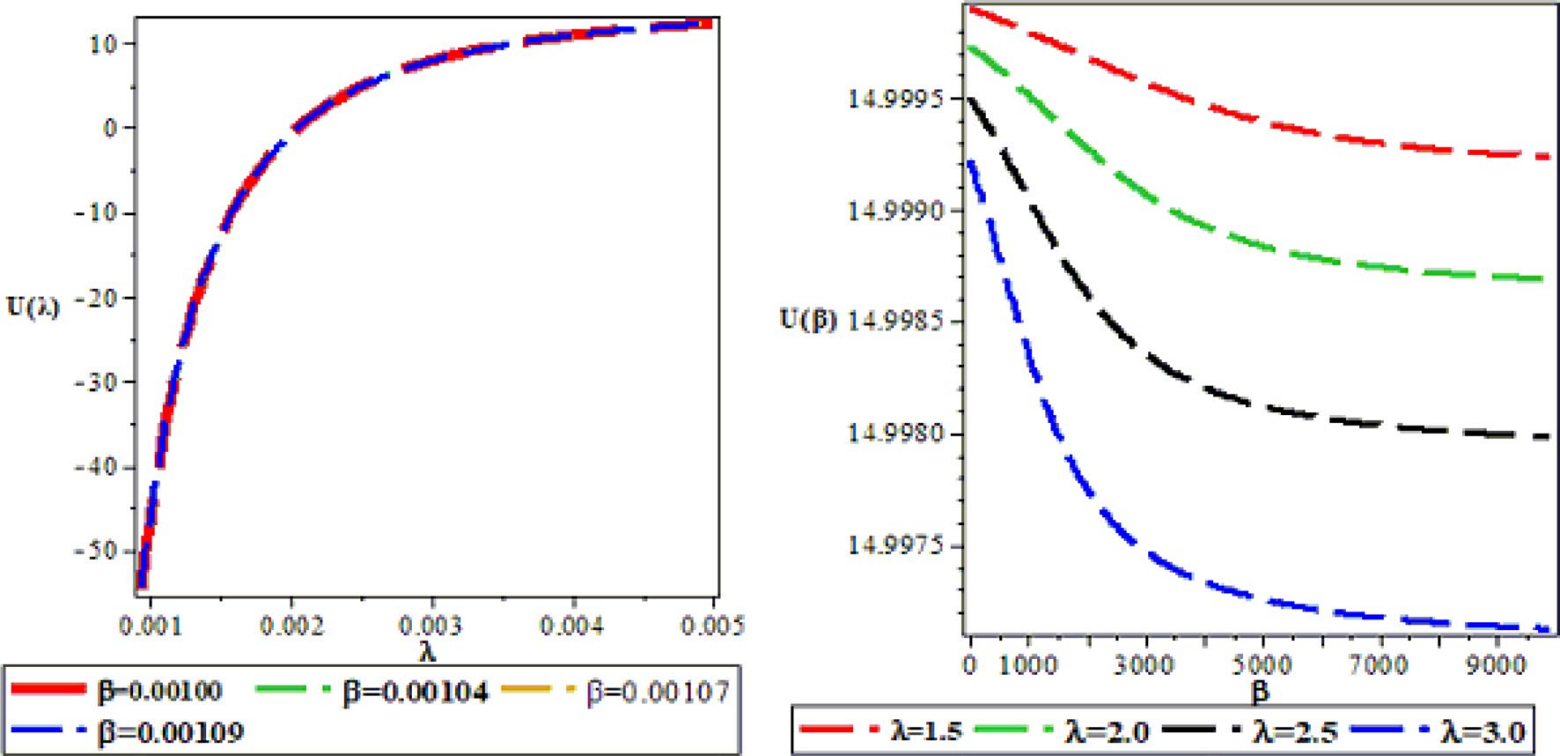
Variation of vibrational mean energy (U) against the maximum quantum state (λ) and the temperature parameter (β) respectively.

**Figure 4:**
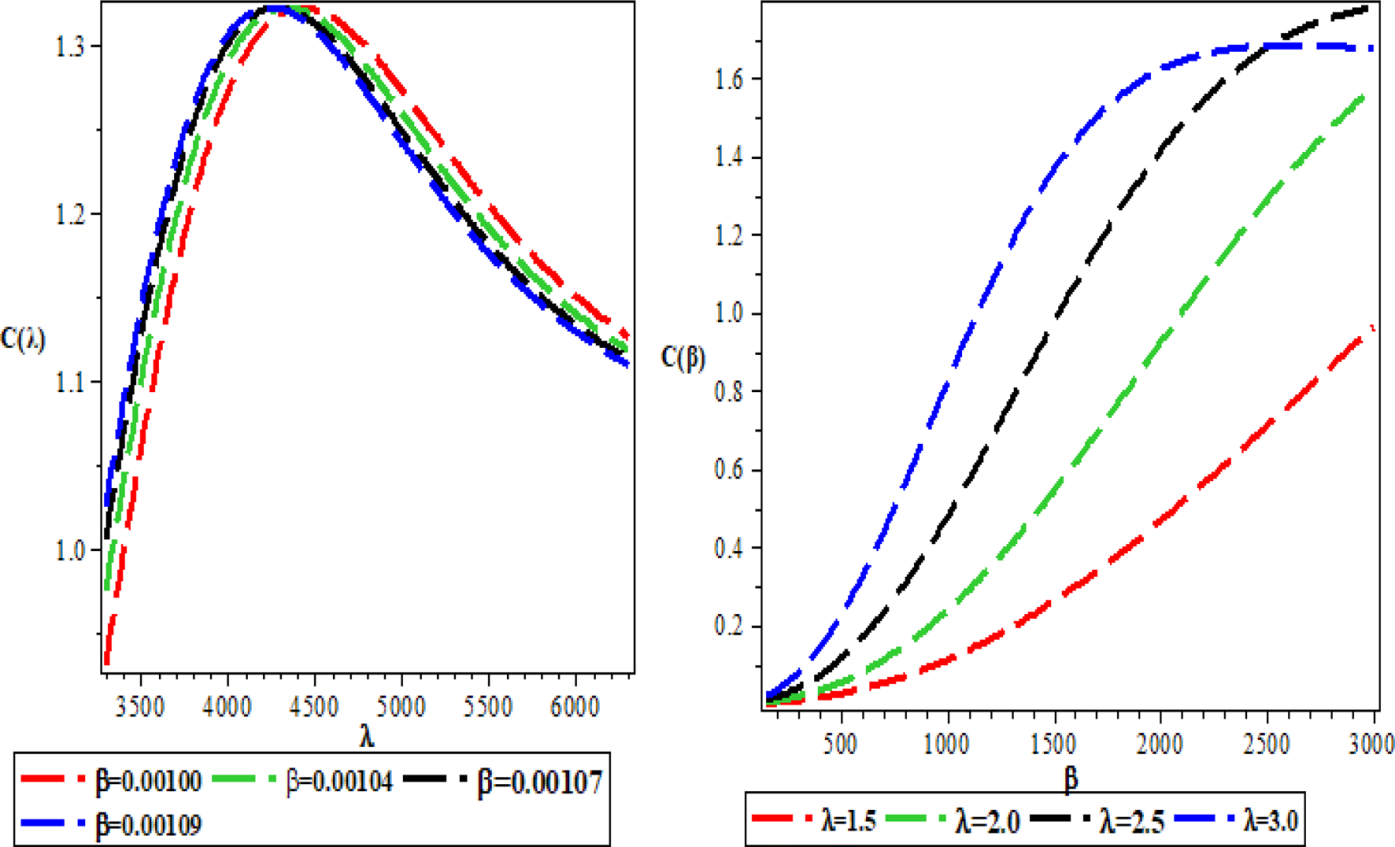
Variation of the vibrational specific heat capacity against the maximum quantum state (λ) and temperature parameter (β) respectively.

**Figure 5:**
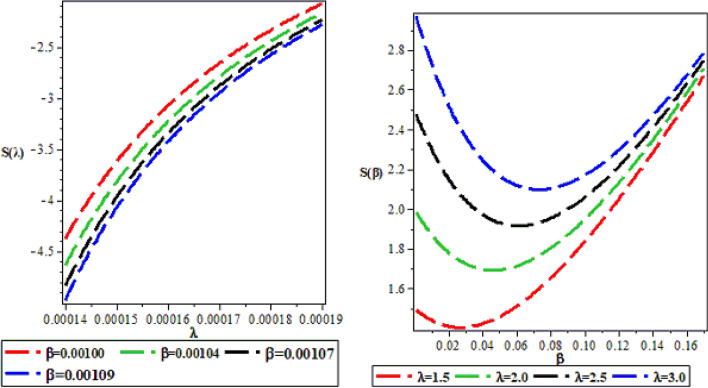
Variation of vibrational entropy against the maximum quantum state (λ) and the temperature parameter (β) respectively.

**Figure 6:**
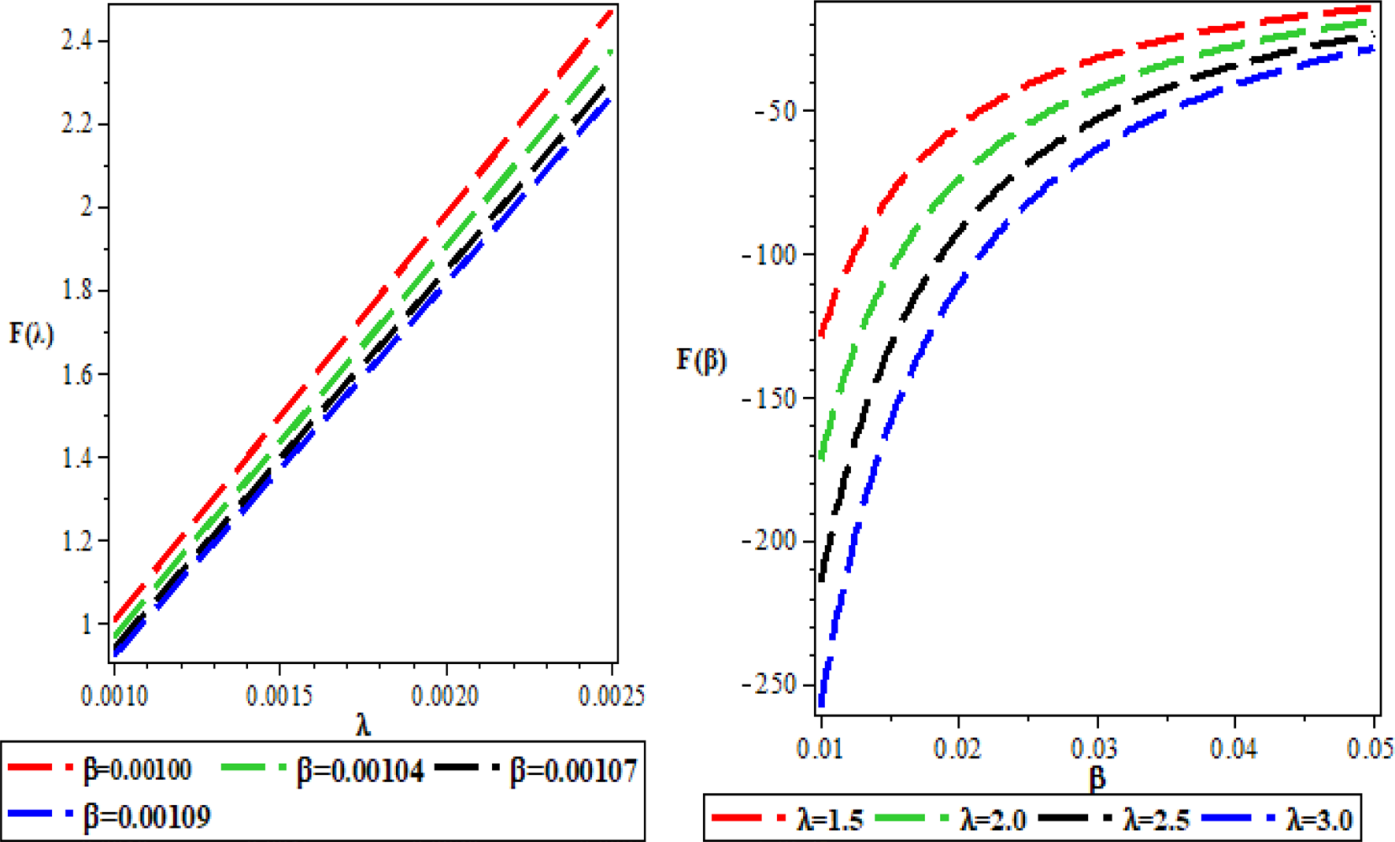
Variation of vibrational free energy against the maximum quantum state (λ) and temperature parameter (β) respectively.

The effects of the three potential parameters on the energy eigenvalues are shown in Fig. 1. It is observed that as $A$ and $C$ respectively decreases from − 10 to minus infinity, the energy tends to be constant. In the same way, as each of $A$ and $C$ increases from 0 to infinity, the energy tends to be constant. In each case, the energy has two turning points. The two turning points lies between − 6.5 and − 4.5 for each of $A$ and $C$. The variation of energy with $B$ goes differently from that of $A$ and $C$. The energy of the system goes to negative infinity as $B$ rises. The energy also has two turning point between $B = - 11.5$ and $B = - 9.5.$ The behaviour of the vibrational partition function with the maximum quantum state (λ) and the temperature parameter (β) respectively, are shown in Fig. 2. The vibrational partition function decreases monotonically as both λ and β respectively, increases. When the partition function decreases as β increases, it simply means that the partition function increases only when the temperature of the system rises. In both cases, the partition function tends to converge at various values of β and λ. In Fig. 3, the plots of the vibrational mean energy against λ and β respectively are shown. The vibrational mean energy rises with an increase in λ but decreases with increasing β. The vibrational mean energy for β = 0.001, 0.00104, 0.00107 and 0.00109 are equal for all values of λ. For various values of λ, the vibrational mean energy tends to converge as the temperature of the system rises gradually. The effects of the maximum quantum state and temperature parameter respectively, are shown in Fig. 4. The vibrational specific heat capacity varies directly with β. At β = 0, the vibrational specific heat capacity for λ = 1.5, 2, 2.5 and 3 converged. As β increases, the vibrational specific heat capacity for various λ diverged. The vibrational specific heat capacity rises as λ increases for some values and have a turning point at λ = 4225. Though the specific heat capacity decreases as λ increases above 4225, the decrease is not as sharp as the increase for λ < 4225. Figure 5 showed the variation of entropy with both maximum quantum state and temperature parameter. The vibrational entropy rises with the maximum quantum state λ for the four values of β studied. In the case of β, the vibrational entropy decreases for some values of β and have a turning point. The turning point of the entropy for various λ differs from one another. However, as the temperature parameter increases, the vibrational entropy for various λ tends to converge. Figure 6 presents the variation of free energy with both λ and β respectively. The vibrational free energy varies directly with the maximum quantum state (λ) and increases monotonically as the temperature parameter (β) increases. At higher values of β, the vibrational energy has a turning point.

Since the potential parameters ($$A$$, $$B$$ and $$C$$) are not spectroscopic parameters, a careful selection of their values is required. To obtain a desired result, in this work, the potential parameters are taken as $$A = \frac{{\lambda_{0} }}{2},$$ $$B = 2(1 - \lambda_{0} )$$ and $$C = A + B + 2$$ in the computations of numerical values. In Table 1, the energy spectrum for three values of the dissociation energy with various quantum number and angular momentum quantum number were presented. As it can be seen from the table, the higher the dissociation energy, the higher the energy of the system. It also shows that the energy of the system varies directly with the quantum number. With a carefully selection of the relationship for the potential parameters, the results obtained for the molecular attractive potential equal the results for Generalized Morse potential. In Table 2, we presented a comparison of the results of the molecular attractive potential and that of the Generalized Morse potential for two different values of the equilibrium bond separation. The results showed a good agreement. In the computations, $$\lambda_{0} = 2.$$ In Table 3, the calculated RKR vibrational energies and the experimental data for cesium dimer and lithium dimer are presented using Eq. (12) and the experimental data taken from Refs.^25^ and^26^. For cesium dimer, $$D_{e} = 2722.28\;{\text{cm}}^{ - 1} ,$$$$r_{e} = 5.3474208\;{\dot{\text{A}}},$$ and $$\omega_{e} = 28.8918\;{\text{cm}}^{ - 1} .$$ For lithium dimer, $$D_{e} = 2722.28\;{\text{cm}}^{ - 1} ,$$ $$r_{e} = 4.173\;{\dot{\text{A}}}$$ and $$\omega_{e} = 65.130\;{\text{cm}}^{ - 1} .$$ The deviation $$\sigma$$ for both the cesium dimer and the lithium dimer were calculated. For the cesium dimer, the deviation increases from the lowest vibrational level to the highest vibrational level. The deviation in the lithium dimer increases from the lowest vibrational level to the first three lower vibrational level, then the pattern of deviation changes without following a definite direction. To ascertain the closeness of the calculated values to the experimental values, the average deviation for each molecule was calculated using the formula^9^

29 $$\sigma_{av} = \frac{100}{N}\mathop \sum \limits_{v} \left| {\frac{{E_{RKR} - E_{v0} }}{{E_{RKR} }}} \right|,$$

where $E_{RKR}$ is the experimental values, $E_{v0}$ is the calculated value and $N$ is the number of experimental RKR data points. With the formula, the average deviation for the cesium dimer is 0.4415% and that of the lithium dimer is 0.0007%.

**Table 1:** Energy spectrum $(E_{n,\ell } )$ for various $n$ and $\ell$ states for three different values of the dissociation energy with $\mu = \hbar = 1,$ $\alpha = 0.25,$ $r_{e} = 0.20$ and $\lambda_{0} = 2.$

$n$	$\ell$	$D_{e} = 5$	$D_{e} = 10$	$D_{e} = 15$
0	0	3.977598297	6.798287056	9.109637217
1	0	4.792524933	9.075571808	13.01290785
	1	4.955213995	9.592230252	13.92388300
2	0	4.967085778	9.696564702	14.20710054
	1	4.999752546	9.882375036	14.57263827
	2	4.980612046	9.981247750	14.83593431
3	0	4.999955612	9.920455196	14.69371353
	1	4.980876842	9.984079176	14.85561791
	2	4.926380148	9.998356127	14.96591760
	3	4.844354168	9.962890521	14.99996366
4	0	4.975269344	9.993868287	14.90806828
	1	4.926609948	9.997542268	14.97297704
	2	4.847057320	9.962692496	14.99999958
	3	4.744069524	9.891525886	14.97589134
	4	4.620971626	9.791084240	14.90996055
5	0	4.916959544	9.991867929	14.98978447
	1	4.847020680	9.959496312	14.99972101
	2	4.746922555	9.891084068	14.97436300
	3	4.625214934	9.792547351	14.90959415
	4	4.484544594	9.668886423	14.81149912
	5	4.326164617	9.523261130	14.68535808

**Table 2:** Comparison of energy eigenvalues as a function of the parameter a for 2*p*, 3*p*, 3*d*, 4*p*, 4*d* and 4*f* states with $\mu = \hbar = 1,$ and $\lambda_{0} = 2.$

State	$\alpha$	Present	^30^	^31^	Present	^30^	^31^
2*p*	0.05	7.860596160	7.86080	7.862800	4.140678930	4.14068	4.14208
	0.10	7.952471120	7.95247	7.955370	4.218346794	4.21835	4.22040
	0.15	8.043224870	8.04322	8.047240	4.295518199	4.29552	4.29870
	0.20	8.132870439	8.13287	8.138420	4.372213175	4.37221	4.37690
3*p*	0.05	10.98907607	10.9976	10.99980	7.524809220	7.53258	7.53500
	0.10	11.14518564	11.1617	11.16470	7.708790323	7.72393	7.72710
	0.15	11.29818128	11.3224	11.32647	7.891196980	7.91330	7.91770
	0.20	11.44806887	11.4795	11.48513	8.072028091	8.10071	8.10660
3*d*	0.05	10.21535527	10.2154	10.21651	5.739126228	5.73913	5.74040
	0.10	10.35103947	10.3510	10.35409	5.843270280	5.84327	5.84650
	0.15	10.48372939	10.4837	10.48992	5.945053133	5.94505	5.95150
	0.20	10.61346374	10.6135	10.62403	6.044533598	6.04453	6.05530
4*p*	0.05	12.48657121	12.4974	12.49920	9.601425934	9.61280	9.61560
	0.10	12.67524352	12.6960	12.69851	9.860761187	9.88269	9.88620
	0.15	12.85677735	12.8865	12.89010	10.11501518	10.1467	10.1514
	0.20	13.03115169	13.0689	13.07400	10.36412165	10.4047	10.4111
4*d*	0.05	12.09143796	12.0977	12.09890	8.485907020	8.49272	8.49480
	0.10	12.27048800	12.2825	12.28570	8.691367758	8.70461	8.70870
	0.15	12.44340752	12.4608	12.46715	8.892880024	8.91218	8.91940
	0.20	12.61023933	12.6326	12.64324	9.090508156	9.11551	9.12720
4*f*	0.05	11.81953623	11.8195	11.82090	7.433455810	7.43346	7.43510
	0.10	11.99296121	11.9930	11.99810	7.581418806	7.58142	7.58680
	0.15	12.16044661	12.1604	12.17180	7.724482273	7.72448	7.73610
	0.20	12.32207217	12.3221	12.34210	7.862757512	7.86276	7.88310

**Table 3:** Comparison of RKR data $({\text{cm}}^{ - 1} )$ with calculated energies of $Cs_{2} \left( {3^{3} \sum_{g}^{ + } } \right)$ molecule and ${}^{7}Li_{2} \left( {a^{3} \sum_{u}^{ + } } \right)$ molecule.

$v$	$Cs_{2}$			${}^{7}Li_{2}$		
	RKR^32^	Calculated values	$\sigma$	RKR^33^	Calculated values	$\sigma$
0	19,477.5507	19,477.55890	− 0.008200	31.8570	31.80133823	0.05566177
1	19,506.2939	19,506.29961	− 0.005710	90.4530	90.39141775	0.06158225
2	19,534.8916	19,534.87673	0.014870	142.523	142.4158566	0.10714340
3	19,563.3470	19,563.29044	0.056560	188.240	188.1839027	0.05609730
4	19,591.6634	19,591.54092	0.012248	227.679	227.4610980	0.21790200
5	19,619.8441	19,619.62831	0.215790	260.837	260.5359641	0.30103590
6	19,647.8922	19,647.55284	0.339360	287.665	287.3994373	0.26556270
7	19,675.8110	19,675.31463	0.496370	308.098	307.9456174	0.15238260
8	19,703.6037	19,702.91391	0.689790	322.155	322.2954606	− 0.14046040
9	19,731.2736	19,730.35084	0.922760	330.170	330.7598462	− 0.58984620
10	19,758.8239	19,757.62551	1.198800	333.269	333.5574895	− 0.02884895

## Conclusion

The solutions of a one-dimensional Schrödinger equation were obtained under a molecular potential with three different potential parameters. It was noted that for negative values of the potential parameters, the potential parameters have the same effects on the energy eigenvalues but for positive values of the potential parameters, the effect of one differs from the effects of the other two parameters. The effect of λ and β on the thermal properties are the same except that of the mean energy. The RKR of both cesium dimer and lithium dimer calculated aligned with the observed values. However, the average deviation of lithium dimer from the observed value is far smaller than the average deviation of cesium dimer from the observed value.

## References

[1] Song XQ, Wang CW, Jia CS (2017). Thermodynamic properties for the sodium dimer. Chem. Phys. Lett..

[2] Liu JY, Hu XT, Jia CS (2014). Molecular energies of the improved Rosen–Morse potential energy model. Can. J. Chem..

[3] Hua XT, Liu JY, Jia CS (2013). The state of Cs2 molecule. Comput. Theor. Chem..

[4] Jia CS, Diao YF, Liu XY, Wang PQ, Liu JY, Zhang GD (2012). Equivalence of the Wei potential model and Tietz potential model for diatomicmolecules. J. Chem. Phys..

[5] da Silva ML, Guerra V, Loureiro J, Sá PA (2008). Vibrational distributions in N2 with an improved calculation of energy levels using the RKR method. Chem. Phys..

[6] Tang HM, Liang GC, Zhang LH, Zhao F, Jia CS (2014). Molecular energies of the improved Tietz potential model. Can. J. Chem..

[7] Zhang LH, Li XP, Jia CS (2011). Approximate Solutions of the Schrödinger equation with the Generalized Morse potential model including the centrifugal term. Int. J. Quant. Chem..

[8] Onate CA, Ikot AN, Onyeaju MC, Ebomwonyi O, Idiodi JOA (2018). Effect of dissociation energy on Shannon and Rényi entropies. Karbala Int. J. Mod. Sci..

[9] Falaye BJ, Oyewumi KJ, Ikhdair SM, Hamzavi M (2014). Eigensolution techniques, their applications and Fisherʼs information entropy of the Tietz–Wei diatomic molecular model. Phys. Scr..

[10] Horchani R, Al-Kindi N, Jelassi H (2020). Ro-vibrational energies of caesium molecules with the Tietz–Hua oscillator. Mol. Phys..

[11] Desai AM, Mesquita N, Fernandes V (2020). A new modified Morse potential energy function for diatomic molecules. Phys. Scr..

[12] Horchani R, Jelassi H, Ikot AN, Okorie US (2020). Rotation vibration spectrum of potassium molecules via the improved generalized Pöschl–Teller oscillator. Int. J. Quant. Chem..

[13] Jia CS, Zhang LH, Wang CW (2017). Thermodynamic properties for the Lithium dimer. Chem. Phys. Lett..

[14] Onate CA, Idiodi JOA (2016). Fisher information and complexity measure of generalized morse potential model. Commun. Theor. Phys..

[15] Oyewumi KJ, Falaye BJ, Onate CA, Oluwadare OJ, Yahya WA (2014). Thermodynamic properties and the approximate solutions of the Schrödinger equation with the shifted Deng–Fan potential model. Mol. Phys..

[16] Qiang WC, Li K, Chen WL (2009). New bound and scattering state solutions of the Manning–Rosen potential with the centrifugal term. J. Phys. A..

[17] Idiodi JOA, Onate CA (2016). Entropy, Fisher information and variance with Frost–Musulin potential. Commun. Theor. Phys..

[18] Onate CA, Onyeaju MC, Ituen EE, Ikot AN, Ebomwonyi O, Okoro JO, Dopamu KO (2018). Eigensolutions, Shannon entropy and information energy for modified Tietz–Hua potential. Indian J. Phys..

[19] Edet CO, Okorie US, Osobonye G, Ikot AN, Rampho GJ, Sever R (2020). Thermal properties of Deng–Fan–Eckart potential model using Poisson summation approach. J. Math. Chem..

[20] Williams BW, Poulios DP (1993). A simple method for generating exactly solvable quantum mechanical potentials. Eur. J. Phys..

[21] Hamzavi M, Eshghi M, Ikhdair SM (2012). Effect of tensor interaction in the dirac-attractive radial problem under pseudospin symmetry limit. J. Math. Phys..

[22] Eshghi M, Hamzavi M (2012). Spin symmetry in dirac-attractive radial problem and tensor potential. Commun. Theor. Phys..

[23] Ita BI, Ikeuba AI, Hitler LM, Tchoua P (2015). Solutions of the Schrodinger equation with inversely quadratic Yukawa plus attractive radial potential using Nikiforov–Uvarov method. IJTPC.

[24] Onate CA, Adebimpe O, Lukman AF, Adama IJ, Okoro JO, Davids EO (2018). Approximate eigensolutions of the attractive potential via parametric Nikiforov–Uvarov method. Heliyon.

[25] Nikiforov AF, Uvarov VB (1998). Special Functions of Mathematical Physics.

[26] Tezcan C, Sever R (2009). A general approach for the exact solution of the Schrödinger equation. Int. J. Theor. Phys..

[27] Ita B, Louis H, Magu TO, Nzeata-Ibe N (2017). Bound state solutions of the Schrӧdinger equation with Manning–Rosen plus a class of Yukawa potential using Pekeris-like approximation of the Coulomb term and parametric Nikiforov–Uarov. Phys. Sci. Int. J..

[28] Ikhdair SM, Hamzavi M (2014). Approximate solutions to spetially-dependent mass Dirac equation for modified Hylleraas plus Eckart potential with Yukawa potential as a tensor. Indian J. Phys..

[29] Rajabi AA, Hamzavi M (2013). A new Coulomb ring-shape potential via generalized parametric Nikiforov–Uvarov method. J. Theor. Appl. Phys..

[30] Dong SH, Gu XY (2008). Arbitrary *l*state solutions of the Schrödinger equation with the Deng–Fan molecular potential. J. Phys. Conf. Ser..

[31] Lucha W, Schöberl FF (1999). Solving the Schrӧdinger equation for bound states with MATHEMATICA 3.0. Int. J. Mod. Phys. C.

[32] Mesa ADC, Quesne C, Smirnov YF (1998). Generalized Morse potential: Symmetry and statellite potentials. J. Phys. A..

[33] Linton C, Martin F, Ross AJ, Russier I, Crozet P, Yiannopoulou A, Li L, Lyyra AM (1999). The hifh-lying vibrational levels and dissociation energy of the state of. J. Mol. Spectrosc..

